# Transurethral resection of bladder tumor-based bladder preservation therapy for refractory high risk non-muscle invasive bladder cancer: Current landscape and future directions

**DOI:** 10.3389/fsurg.2023.1143219

**Published:** 2023-04-12

**Authors:** Xinghui Sun, Tianzeng Dai, Lihui Xu

**Affiliations:** ^1^ Department of Urology, Mengchao Hepatobiliary Hospital, Fuzhou, China; ^2^ Department of Urology, Fujian Medical University, Fuzhou, China

**Keywords:** bladder cancer, non-muscle invasive bladder cancer (NMIBC), recurrence, TURBT (trans-urethral resection of bladder tumor), urothelial cancer, immunotherapy

## Abstract

Bladder cancer is the most common malignant tumor of urinary system worldwide. Approximately 75% of patients with bladder cancer present with non-muscle-invasive bladder cancer (NMIBC), which is effectively managed with transurethral resection of bladder tumor (TURBT). For refractory high risk NMIBC, patients are typically treated by radical cystectomy (RC). TURBT deserves further evaluation. Growing evidence suggests that repeated TURBT-based bladder-sparing approaches may improve oncological outcomes and quality of life in highly selected patients. Novel imaging techniques and biomarkers may aid in patients selection and postoperative surveillance. With growing interest in adding immunotherapy to refractory bladder cancer, TURBT based approaches enable the bladder preservation therapy for high risk NMIBC. Here we summarize the current landscape, biomarkers for surveillance, and future directions for applying TURBT-based bladder preservation therapy.

## Introduction

It is estimated that bladder cancer is the second most common urological cancer in the world (1). Approximately 75% of patients with bladder cancer present with non-muscle-invasive bladder cancer (NMIBC) (2). As of now, NMIBC patients are classified into low-, intermediate-, high and very high-risk categories according to their tumor stage, grade and the risk of progression or metastasis (3). Patients with low- or intermediate-risk NMIBC are generally treated with transurethral resection of bladder tumor (TURBT) associated or not with intravesical therapy, but selecting an ideal therapy for refractory high risk NMIBC remains a matter of debate. With the emergence of bladder-preservation strategies based on a combination of complete TURBT, chemotherapy and immunotherapy and novel imaging techniques and biomarkers for patient selection, repeated TURBT for recurrent and high risk NMIBC deserves further evaluation. Here, we summarize the current landscape, biomarkers for surveillance, and future directions of bladder preservation therapy for refractory high risk NIMBC are also discussed.

### Radical cystectomy or repeated TURBT for recurrent NMIBC, that is a question

The treatment of refractory high risk NMIBC involves TURBT with intravesical bacillus Calmette-Guérin (BCG) or immediate radical cystectomy (RC). As refractory/recurrent NMIBC has higher risk of recurrence and progression rate, RC is generally selected at the recurrence of NMIBC. Patients treated with RC may have a favorable oncological outcome. Wan JCM (4) conducted a systematic review, total 10 studies from 1946 to 2020 were selected, including 1,516 patients with high grade NMIBC who underwent either primary RC or delayed RC. It was found that early RC improves the 5-year and 10-year cancer specific survival (CSS) compared to delayed RC. Another study reported that patients staged as cT1 treated with primary RC had improved 5-year CSS compared to patients treated with TUBRT plus intravesical BCG (100% vs. 60% *p* = 0.006) and patients who underwent delayed RC after recurrence (62%, *p* = 0.015). An average follow-up of 67.5 months revealed 75%, 45%, and 35% of those treated with intravesical BCG experienced disease recurrence, progression, and lymph node metastasis, respectively (5). On the other hand, it was found that about 40%–50% of tumors in stage T1 NMIBC at TURBT are upstaged to MIBC at RC (6–8). Retrospective data on 3,393 patients collected from nine centers showed up to 45.9% (*n* = 592) of 1,291 NMIBC patients progressed to MIBC (6). Patients experienced the progression of NMIBC to MIBC have lower 10-year recurrence-free survival (36% vs. 43%, *P* = 0.01), CSS (37% vs. 43%, *P* = 0.01) and over survival (28% vs. 35%, *P* = 0.01) than those MIBC patients without a history of NMIBC (9).

In contrast, it was noted that RC did not have a superiority in terms of oncological outcomes and postoperative complications in other reports. In an earlier respective study, immediate RC at the diagnosis of NMIBC were performed in 134 patients and delayed RC were performed in 70 patients after the recurrence of bladder preservation therapy. The survival of NMIBC patients, 3-year (84% vs. 79%), 5-year (78% vs. 71%) and 10-year (69% vs. 64%), was statistically similar after primary and delayed RC (10). RC may decrease the risk of recurrence and progression of refractory high risk NMIBC but may be associated with perioperative or postoperative complications and reduction in quality of life. One long-term study revealed there were 28.0% of RC-related perioperative complications and 2.5% of RC-related perioperative deaths (11).

Additionally, clinicians and NMIBC patients face the uncertainty of potential under- or over-treatment. Some studies reported that around one-fourth patients who had TURBT-based MIBC tumor down staged to NMIBC after RC (12). Dyer et al. (13) compared the pathological findings of same patients with bladder cancer who had initial TURBT and subsequent RC, finding that among the 1,039 cases with T2 + disease at TURBT, 106 (10%) had been down-staged to < T2 at RC. Metastasis of NMIBC to distant sites without evidence of progression or regional metastasis is infrequent. The patients with tumor downstaging at RC should have a long-term survival rates and better quality of life before progressing to MIBC or never occur. For refractory high risk NMIBC, RC could represent an over-treatment of many NMIBC patients who may not ultimately progress to disease.Studies concerning the quality of life, the morbidity and mortality associated with RC have attracted increased level of interest in bladder preservation strategies, the impact of RC deserves further evaluation.

A multi-institution analysis indicated that salvage intravesical therapy after BCG had not increase the upstaging risk and decrease the CSS of patients with high-grade NMIBC who ultimately underwent RC (14). In this respective study, of 378 patients with high risk NMIBC, salvage intravesical therapy were underwent in 62 cases, the 5-year CSS were 73% for salvage intravesical therapy and 74% for no.On multivariable analysis, patients received salvage intravesical therapy was not significantly associated the risk of death from bladder cancer (HR 1.12; 95% CI 0.60–2.09, *P* = 0.7) (14).

Peilin and his colleagues (15), recently, used meta-analytic methods to analyze 11 cohorts studies of 1,735 patients with high grade NMIBC. The meta-analysis documented that mixed bladder preservation modalities had a better effect on over survival than RC and a worse effect on CSS with low evidence strengths. Among patients older than 65 years, bladder preservation had better 2-year, 5-year, and 10-year over survival than RC. Bladder preservation is a superior treatment option in high grade NMIBC compares to RC, especially for older patients.

A study from real-world reported the different oncological outcomes between RC and bladder preservation for early relapse and refractory NMIBC. There was no significant difference between the RC and non-RC groups in CSS after matched score analyses (16).

Based on these contradictory results, the optimal individual treatment for refractory NMIC must be selected based on their condition. TURBT based bladder preservation which can improve the accuracy of cancer staging and facilitate the selection of the best treatment for the NMIBC patient deserves further evaluation.

### If TURBT is completed, why the disease still progress?

TURBT is the standard procedure for the evaluation and management of NMIBC. However, even with the best treatments, involving complete TURBT, subsequent intravesical therapies and extended surveillance, recurrence and progression can still occur in high-risk NMIBC. If TURBT is completed, why the disease still progress? Though having no systematic study, there are likely a few explanation in light of previous studies and clinical experience.

Firstly, lymphovascular invasion in TURBT specimens is intimately related to an increased risk of pathological upstaging and a poor clinical outcomes in NMIBC tumors (17, 18). Even after resection of all visible tumors, approximately one third of lesions had residual tumor at the base and periphery (19). A systematic review including 8,409 patients from 31 studies reported that the residual tumor tissues were found in 17%–67% of the patients with high-grade Ta tumor and in 20%–71% of the patients with T1 stage tumor by repeated TURBT, respectively (20). The results of Gill et al. (21) study indicated that the incidence of residual tumor was 44.2% in NMIBC patients at initial TURBT and the incidence of residual tumor was positively correlated with tumor upstaging. The absence of detrusor muscle is associated with a higher risk of residual disease and early relapse. The real completed TURBT should have no residual tumor in their repeated TURBT specimens.

Secondly, the presence of a multifocal tumor is also a high risk factor for NMIBC recurrence and progression. In a predicting recurrence study of 477 patients with NMIBC, 365 patients had multifocal tumors in the initial TURBT, of them, 239 (73.1%) had recurrence. Based on the results of Cox regression analyses and prediction model, multifocal tumors is an important risk factor affecting NMIBC recurrence (22).

Thirdly, tumor dissemination was generally found after partial cystectomy (23). Intravesical dissemination of NMIBC during TURBT is rarely reported or studied. While immediate intravesical chemotherapy or BCG immunotherapy is routinely recommended by guidelines and can decrease recurrence and progression of NMIBC, intravesical dissemination of bladder tumor during TURBT still need to draw our attention (3).

Fourthly, histology concordance between TURBT and RC is little studied. The reason, on the one hand, is considerably less patients with bladder cancer who had initial TURBT and subsequently RC in the same hospital; on the other hand, is that urologist lack sufficient awareness of the histological variants between TURBT and RC (24). The pathological findings of 1,580 patients who had initial TURBT and subsequent RC were analyzed by Dyer et al. (13). The histopathology report found no indication of muscularis propria invasion in 34% of cases at time of TURBT, but at time of RC, 56% were upstaged to T2 + disease. Of 132 patients with *in situ* carcinoma (CIS) at TURBT, 90 (68%) were turned out to be MIBC at RC. It is also interesting to note that about 27%–51% of pT1 tumors diagnosed with TURBT were upstaged to MIBC at RC in other studies (7, 8). Ngo et al. (25) retrospectively analyzed 110 patients with bladder cancer who underwent TURBT initially and subsequent RC, discordant histology between TURB and RC was found in 18% of the patients. Discrepancies can be seen between TURB and RC in NMIBC, accordingly, RC is indicated for patients with refractory high NMIBC (3). The International Consultation on Cancer Reporting (www. ICCR-cancer.org) and the WHO 2016 require reporting of histological variants and their percentages in the pathology report. Due to the histological variants, TURBT alone is not sufficient in most high risk NMIBC.

### Also raised is the issue of how to judge whether TURBT was effective

The results of a TURBT are considered successful when all tumor tissue has been removed and the resection margins of the specimen are cancer free and the detrusor muscle is presented in the specimen. But the presence of detrusor muscle in tumor specimen just contributed to the decision to either accept or decline repeated TURBT. Currently, we have no enough evidence to determine whether TURBT is effective. New technologies for improved cystoscopy are being developed to visualize the residual tumors that are present but not visible under white light cystoscopy. Narrow-band imaging (NBI) cystoscopy and fluorescence guided TURBT are recommended. Fluorescence guided TURBT was shown to be effective in delaying recurrence and progression (26). A systematic review including 17 prospective trials indicated that combination of photodynamic diagnosis fluorescence guided TUBRT and NBI cystoscopy decreased recurrence rates and increased the visualization and detection of residual tumors compared to white light cystoscopy alone (27). However, the results of NBI cystoscopy on detection of residual tumors are inconsistent and inconclusive (26, 28). In a randomized controlled trials, the recurrence rates of 294 patients underwent NBI guided TURBT and 303 patients received conventional TURBT were 27.1% and 25.4%, respectively. The one-year recurrence rate of NMIBC in NBI guided TURBT was similar to that in conventional TURBT. Similar conclusions were given by another recent meta-analysis (29). Nevertheless, the follow-up period in both studies was relatively short. Long-term results could be different (28).

### Surveillance

Early detection of recurrence plays a crucial role in the bladder preservation. When recurrence is detected, repeated TURBT and additional adjuvant therapies including intravesical BCG, chemotherapy and immunotherapy continue to contribute to bladder preservation. Nevertheless, the poor precision in identifying which patients with refractory NMIBC should be offered TURBT with BCG or immediate RC remains a major challenge.

Undoubtedly, the basis of active surveillance in bladder cancer is regular cytology and cystoscopy (30). Several biomarkers have been linked to recurrence and progression and proposed for NMIBC surveillance. SOX4 might potentially serve as a biomarker for predicting the underlying progression from NMIBC to MIBC (31). A significant difference in epidermal growth factor-containing fibulin-like extracellularmatrix protein 1 (EFEMP1) expression exists between MIBC and NMIBC, indicating its association with the muscle invasion and it can predict a high bladder recurrence rate after adjusting for tumor stage and grade (32). The expression of the let-7c cluster in the urine was independently associated with progression of bladder cancer at higher T-stages.The evaluation of urinary let-7c clusters may improve prognoses by identifying patients who are at risk of progression and addressing them early with radical treatments (33). A marker for TP53 mutational status could be used to predict recurrence and prognosis in the early stages of NMIBC (34). The identification of accurate and novel biomarkers will improve treatment options and surveillance methods for NMIBC.

### Future directions

Future work should aim to detect and recognize those tumors with high risk of recurrence. Bladder tumor markers for prediction of recurrence and progression and intend to replace urinary cytology or cystoscopy is currently under investigation. Precision testing of residual tumors or upstaging is the first and most important prerequisite for the choice of surgical procedure. Detecting invisible residual tumors in deep tissue and suspicious lesions decreases the incidence of tumor recurrence (17). Next generation sequencing (NGS) can provide nearly complete information of human genetic mutation and is used to identify the tumor markers. Using NGS study, NEB, MLH1, GATA3, FGFR1 and RAF1 et al., 18 genes were found correlated with recurrence of NMIBC (35). It was found that NEB, FGFR1 and SDHC were independent prognostic predictor for recurrence in patients treated with BCG. Surveillance of tumor mutation burden using NGS could provide predictor for recurrence.Targeted sequence analysis of ctDNA is also a promising approach to predicting disease recurrence in patients with NMIBC after TURBT and immunotherapy (36).

Numerous studies and risk classification models have been developed to predict NMIBC recurrence and progression.The EORTC risk tables is a well-known model for prediction of the risk of NMIBC recurrence and progression (37). More risk classification models based on prognostic factors are expected to increase the accuracy of identifying patients at high risk.

Cancer treatment has entered a new era thanks to drugs that block immune checkpoints. The immune checkpoint inhibitors are being tested for bladder cancer. Several recent clinical trials have been carried out to evaluate the role of checkpoint inhibitors in bladder cancer and yielded remarkable pathological results (38). The phase 2 PURE-01 trial was designed to evaluate the efficacy of pembrolizumab in neoadjuvant immunotherapy for MIBC. Among the 50 enrolled patients, MIBC downstaging to NMIBC were observed in 54% of patients (39). New immunotherapeutic agents, such as atezolizumab, pembrolizumab, nivolumab, durvalumab, and avelumab are already licensed to treat bladder cancer by the United States Food and Drug Administration (FDA) and have obtained satisfactory results. A new era in bladder cancer treatment has begun with the introduction of perioperative immunotherapy with checkpoint inhibitors.

Different studies reported that the bladder cancer had as many as 13 histological variants, different histological variants vary significantly in their progress and oncological outcomes (40). For example, patients with glandular differentiation are inclined to have higher pathological grade and progression rate (41, 42). Histological variants should be considered to determine the surgical modality (TURBT or RC).

Cancer stem cell are tumor initiating cells and resistant to standard chemotherapeutic and radiation therapy, which are thought to contribute to tumor progression and metastasis. Cancer stem cell was also found in the bladder tumor. The importance of cancer stem cells interaction with their microenvironment in cancer progression has been attracting increasing attention in cancer research. Exosome secreted by stem cells, cancer stem cells and stromal cells mediate the intercellular communication among these cells and modulate cancer progression. On the other hand, exosome-based nanometric vehicles are capable of delivering anti-cancer drugs and genes for cancer stem cell targeting therapy (43). Understanding the biological interactions of exosomes with the tumor microenvironment will facilitate the study of bladder cancer progression, and exploring exosome-based drug delivery system specifically targeting bladder cancer stem cells will offer new therapeutic opportunities for the treatment of bladder cancer. The clinical applicability and effectiveness of exosome-based drug delivery system are being tested in clinical trials.

Antibody-drug conjugates (ADCs) are targeted anti-cancer agents made up of monoclonal antibodies, linkers, and cytotoxic drugs (payloads). In recent years, ADCs have been recognized as an innovative therapeutic approach that can take advantage of tumor-specific molecular characteristics. There are five ADCs approved by FDA for advanced urothelial carcinoma treatment and more ADCs are currently under intensive evaluation for clinical trials (44). The combination of ADCs with TURBT may provide bladder preservation for refractory high risk NMIBC. (new added paragraph as the review advised).

The natural history of NMIBC state has been poorly understood to date. Further investigations are needed to elucidate that recurrent NMIBC is single or multiple, and recurrent multifocal NMIBC is due to tumor dissemination of first TURBT or due to upstaging and upgrade of the residual tumors. The number of recurrences did not appear to be associated with the risk of progression (45). Multiple TaG1 recurrences just increase the risk of subsequent recurrence, is not associated with cancer progression. From my own perspective, it is worthwhile to attempt TURBT for primary multifocal NMIBC tumors instead of RC. Large randomized controlled trials is needed to focus on comparison of third or more repeated TURBT with RC on recurrent NMIBC.

## Discussion

Cancers with stage Ta or T1, as well as carcinoma *in situ* (CIS), are classified as NMIBC. The most effective treatment in these cases is TURBT. There have been significantly growth in the availability of bladder preservation techniques over the past decade with the focus on improving quality of life. Although there have no encouraging randomized controlled trials comparing trimodality treatment to RC, TURBT-based multiple therapy, including perioperative chemotherapy and immunotherapy, ADCs and intravesical BCG, substantially increase the possibility of bladder preservation (46). The diversity and uniqueness of the patients should be take into account, and a combined and individualized treatment may be the best and most effective option.

Bladder preservation may be obtained through early bladder cancer recurrence detection. Various biomarkers in urine or in circulation predict disease recurrence and progression in NMBC. New and developing technologies in cystoscopy, such as NBI cystoscopy and fluorescence guided TURBT provide new opportunities to improve the visualization of the residual tumors.

Based on bladder preservation, future directions should also aim to understand the biological interactions of exosomes with the tumor microenvironment. New anti-cancer drugs based exosome delivery and ADCs may provide novel bladder preservation therapy for refractory NMIBC.

## Data Availability

The original contributions presented in the study are included in the article/Supplementary Material, further inquiries can be directed to the corresponding author.
